# Prevalence of glucose intolerance and metabolic syndrome within one year following delivery of a pregnancy complicated by gestational diabetes

**DOI:** 10.1186/s40834-018-0080-y

**Published:** 2018-11-23

**Authors:** Neetu K. Sodhi, Anita L. Nelson

**Affiliations:** 1 Bloom Obstetrics and Gynecology, 18555 Ventura Blvd, Suite C, Tarzana, CA 91356 USA; 2 Los Angeles BioMedical Research Institute at Harbor UCLA Medical Center, 1457 3rd Street, Manhattan Beach, Torrance, CA 90266 USA

**Keywords:** Gestational diabetes, Metabolic syndrome, Practice guidelines, Longitudinal screening, Cardiovascular risk factors

## Abstract

**Background:**

Women with a history of gestational diabetes (GDM) are at risk for development of both overt Type 2 diabetes (T2DM) and cardiovascular disease (CVD) at higher rates and at earlier ages than control women. Current guidelines recommend longitudinal testing of glucose tolerance for women with prior GDM, but no formal assessments of cardiovascular disease are suggested. This study estimated the prevalence of metabolic syndrome in women with GDM in recent pregnancy who were followed for at least 1 year postpartum to quantify their cardiovascular risks.

**Methods:**

This is a retrospective study of women who were diagnosed with GDM in a public hospital and followed for at least 1 year after delivery and who had tests performed at a minimum 4–12 weeks postpartum and 6 and 12 months postpartum. Primary outcomes were prevalence of glucose tolerance abnormalities and metabolic syndrome (MetS) defined by two prevailing sets of diagnostic criteria.

**Results:**

One hundred fifty-one indigent, primarily Latina women who had been diagnosed in their last pregnancy with GDM comprised the study population. At the first visit postpartum, 4.7% were found to have overt diabetes and between 24 and 31% met the criteria for MetS. By the end of 12 months, another 14.5% were diagnosed with overt diabetes, and 38.5% had prediabetes. An additional 12–25% of the woman who had not had MetS at baseline developed MetS by the end of the 1-year follow-up.

**Conclusions:**

Given the high prevalence of MetS among women with recent history of GDM immediately postpartum and its rapid development in the following year, further research is needed to enable the development of practice guidelines that will define appropriate short and long-term evaluations needed to assess risk for cardiovascular disease in these women.

## Background

Gestational diabetes (GDM) has long been recognized as a strong predictor for the early development of Type 2 diabetes mellitus (T2DM) and cardiovascular disease (CVD) [1–6]. Women with a history of GDM have up to a 70% chance of developing overt diabetes in their lifetimes [1, 7–9]. The risk of cardiovascular disease in women with a history of GDM is at least twice that of women without that history, even after controlling for age, body mass index, smoking, Townsend (deprivation) quintile, baseline lipid-lowering medication and baseline hypertension [3, 6, 10–13]. Some studies show that this increased CVD risk is independent of the development of Type 2 diabetes mellitus [10].

Routine testing for glucose tolerance 4–12 weeks after delivery with a 2-h 75-g oral glucose tolerance test is standard of care following a pregnancy complicated by GDM [9]. Early postpartum testing is done to distinguish women who had undiagnosed pre-existing diabetes from those who only had gestational diabetes [9]. For those with normal oral glucose tolerance test results postpartum, the American College of Obstetricians and Gynecologists (ACOG) recommends assessment of glycemic status every 1–3 years [9]. The American Diabetes Association (ADA) recommends repeat testing of glucose metabolism every 3 years [14].The Endocrine Society calls for “periodic glucose assessment” at unspecified intervals [15].In Sweden, England and other countries, annual repeat testing is recommended [7].

Adherence to postpartum testing guidelines is notoriously poor. Only 50–60% of women diagnosed with GDM, who are seen for postpartum care, are administered any postpartum glucose testing in the year following delivery [6, 16]. Even more do not get tested because they do not return for postpartum care. One recent study found that almost half of all postpartum women are not seen for any postpartum care within 99 days of delivery, even when such services were available for free [17]. Patients may not return for postpartum care because they are anxious about their condition or the visit costs [16]. Low testing rates may also be explained by the fact that significant gaps exist in clinician knowledge and practice relating to postpartum care for women who have had GDM [18].The complexity of the oral glucose tolerance test itself or a failure of clinicians to order the test may also contribute. Inadequate coordination of care between the woman’s obstetrician and her primary care provider has also been identified as a barrier to timely postpartum glucose tolerance testing [6, 16]. This lack of testing is so prevalent that some experts have suggested oral glucose tolerance testing be done while the patient is still hospitalized on postpartum day two [19].

Longer term adherence to screening test recommendations is also not common, even though abnormalities in glucose tolerance can develop rapidly in the months and years following delivery [20]. In a 15-year follow-up study of women with prior GDM, the authors observed that most women did not seek medical care until they developed clinical symptoms of diabetes [7].

Most professional attention has been focused on the high lifetime risks that women with a history of GDM face for developing T2DM. Even though these women are also at high lifetime risk for cardiovascular disease, partial or complete formal testing for cardiovascular risks for women with history of GDM is not routinely recommended by any professional organization [9, 14, 15]. O’Higgins found that women previously diagnosed with gestational diabetes mellitus are not even routinely screened for cardiovascular risk factors [21].Metabolic syndrome, which is commonly used as a marker for cardiovascular risk in the general population, is not listed in any postpartum practice guidelines for women following pregnancy complicated by GDM. The latest guidance documents from both ACOG and ADA make no mention of postpartum CVD risk assessment [9, 14] That lack of direction is reflected in practice; screening for cardiovascular risk factors such a smoking, high body mass index, hypertension and dyslipidemia occurs to be no more often among women who had GDM than it is among control women [6].

In face of the rapid deterioration of glucose tolerance in women with a history of CVD in the months immediately following delivery, it was hypothesized that metabolic syndrome might also develop among women with GDM in their recent pregnancy. This study examines the prevalence of MetS immediately postpartum and within 12 months of delivery. [22].

## Methods

This is a retrospective study of the medical records of women cared for in a clinic at Harbor-UCLA Medical Center, which provided gynecological care to women, who in their last pregnancy had been diagnosed with gestational diabetes using prevailing National Diabetes Data Group criteria at the time of diagnosis. Those criteria included glucose concentrations (fasting and 3 hourly levels following a 100 g oral glucola load) with upper limits normal being 105/190/165/145 mg/dL [23]. Throughout the study period, the protocols for care of women with GDM were stable. The cost of postpartum care for these patients was completely covered for at least 6 weeks postpartum by the pregnancy-only emergency MediCal (California Medicaid) program. Beyond that initial Medicaid-funded postpartum period, many women were enrolled into the California Medicaid-waiver program that provided them free contraceptive services. Others continued to be seen in the clinic under a Los Angeles County ability-to-pay program that was available at the time.

The clinic was overseen by an endocrinologist (diabetologist) and staffed by two women’s health care nurse practitioners who also specialized in gestational diabetes. The patients were scheduled to be seen at or before 6 weeks postpartum and every 3 months thereafter. Laboratory testing was extensive; glucose tolerance tests, lipid panels, C-peptide levels were obtained at least every 6 months. Blood pressure, weight, BMI, waist and hip circumferences were routinely measured at each visit, although patients could decline any measurement. In addition to diabetes counseling, lifestyle promotion and breastfeeding support, social services, trained dietary counseling and contraception were all provided. Once a woman was diagnosed with overt diabetes, her care was transferred to another diabetes clinic.

Approval to conduct this study was obtained from the John F. Wolfe Institutional Review Board and the Research Committee at the Los Angeles BioMedical Research Institute at Harbor-UCLA Medical Center (project number 20547–01). Approval was granted on an exempt basis because it was determined that the risk of harm to the subjects was no greater than minimal and no personal identifiers were to be used.

Data were extracted by the authors directly from the clinic summary sheets that were filled out by the provider at the time of the visit. These sheets recorded for each patient all of the information collected to follow GDM-related outcomes over time. If questions arose, it was possible to consult the medical records for clarification.

Diagnosis of metabolic syndrome (MetS) was made using the two most recognized definitions of “metabolic syndrome” including the National Cholesterol Education Program-Adult Treatment Panel III (NCEP-ATP III) criteria and the International Diabetes Foundation (IDF) criteria to enable comparison with other studies [24, 25]. See Tables 1 and 2 for specific criteria used to define each MetS in each system. Both these criteria consider blood pressure, dyslipidemia and glucose abnormalities and measures of obesity. One difference between NCEP-ATP III and IDF is that the former uses BMI and latter uses waist circumference as a measure of central obesity although BMI > 30 can be used in IDF if waist measurements are lacking. More significantly, NCEP-ATP III allows for any 3 of 5 abnormalities to diagnose MetS, whereas IDF requires central obesity, but uses any 2 of the remaining 4 criteria to complete the diagnosis. Definitions of postpartum diabetes and prediabetes were compatible with the American Diabetes Association’s Diagnosis and Classification of Diabetes Mellitus [14]. The frequencies of abnormal patterns were calculated using descriptive statistics. A *p* value of < 0.05 was selected as the threshold for statistical significance.

**Table 1:**
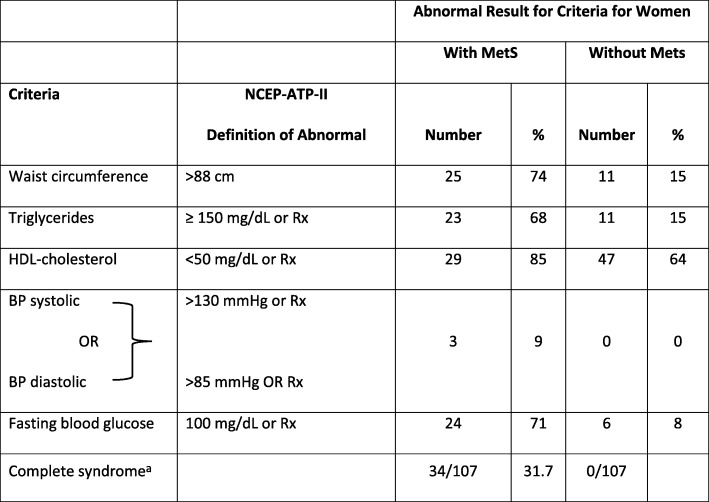
Number and Percent of Women with Abnormal Test Results at 4–12 Weeks Following GDM Delivery using NCEP-ATPIII^b^ Criteria for Metabolic Syndrome (MetS)

^a^Diagnosis requires any 3 of the 5 criteria

^b^*NCEP-ATP III* National Cholesterol Education Program – Adult Treatment Panel III, *Rx* any medication prescribed to treat abnormalities

**Table 2:**
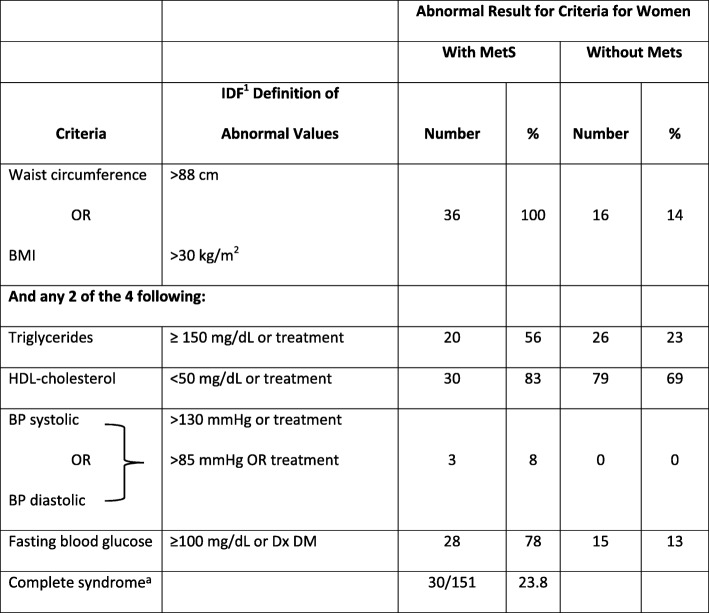
Number and Percent of Women with GDM with Abnormal Test Results at 4–12 Weeks Following Delivery using IDF^a^ Criteria for Metabolic Syndrome (MetS)

^*^Diagnosis requires any 3 of 5 criteria = 30

^a^*IDF* International Diabetes Federation, *Tx* Any specific treatment for condition, *Dx DM* Diagnosis of diabetes

## Results

**Fig. 1:**
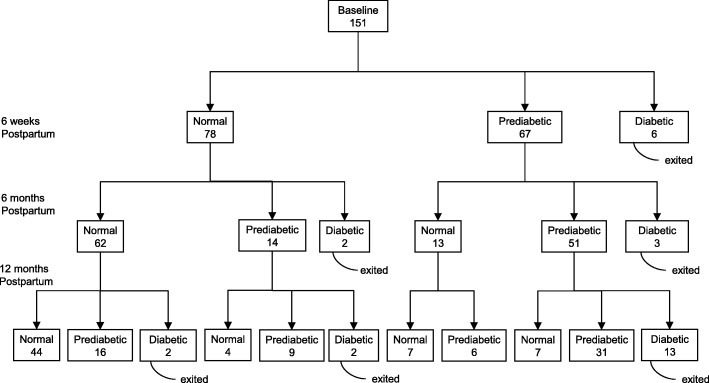
Outcomes of testing for glucose tolerance in women with gestational diabetes in recent pregnancy (*n* = 151)

The study population included the 151 women who had been seen in the postpartum GDM clinic for at least the following 3 visits: at 6-weeks and approximately 6 and 12 months later. The women studied were indigent and primarily Latina, with a mean age of 32 (range of 20–42 years) and a mean parity of 2.6. The mean body mass index (BMI) was 30.0 kg/m^2^ (range 20.0–46.5 kg/m^2^). Figure 1 (entitled Outcomes of testing for glucose tolerance in women gestational diabetes in recent pregnancy (*n* = 151)) displays the frequency with which prediabetes and overt diabetes developed in these women in the first 12 months. At the initial 6 weeks postpartum evaluation, 44.4% had prediabetes and 4.7% were excluded from later analysis because they were diagnosed with overt diabetes. Over the one-year study period, another 14.5% of the remaining subjects developed overt diabetes and another 38.5% who initially had normal glucose tolerance, became prediabetic.

Tables 1 and 2 show the percentage of women who had abnormal values in the variables used to diagnose metabolic syndrome at 4–12 weeks by criteria used for diagnoses of MetS. By that postpartum visit, nearly 1 in 3 postpartum women with recent GDM was diagnosed as having metabolic syndrome by either classification system. The major reason that the numbers of women included in the ATP III group were lower (107) than those in the IDF group (151) is that the latter allowed either BMI or waist circumference to be used as a measure of central obesity. Information about BMI was more readily available for more women than was waist circumference.

The value of considering the syndrome as a whole can be seen in the prevalence of individual abnormalities among those without MetS in Tables 1 and 2. By definition, all women with MetS in the IDF group had central obesity, but 14% of those without MetS by IDF criteria at baseline also had central obesity. By the NCEP-ATP III criteria, a similar pattern was seen; 74% of those with MetS had obesity, but 15% of those without MetS had BMI > 30 kg/m^2^. The other variables were similar between the groups. Abnormal fasting blood glucose was found in 71–78% of subjects with MetS, but 8–13% of those without MetS also had elevated FBS. Low HDL was fairly common in all subjects (83–85% with MetS and 64–69% without MetS), but hypertriglyceridemia was much more prevalent among women with MetS compared to those without MetS (56–68% vs 15–23%). Blood pressure elevations were uncommon in any of our patients. The high rates of abnormalities discovered at the first test cannot be attributed to physiologic changes of pregnancy, since, in general, they persisted throughout the 12-month follow-up period, with the exception of hypertriglyceridemia.

Prevalence of MetS increased in our subjects over time. See Table 3. By 12 months, of the women who did not have MetS initially, 18 (24.7%) met the ATP III criteria for MetS by 12 months, 14 (11.6%) met the IDF criteria. For those women who developed MetS, elevated FBS was the most frequent element to change, but hypertriglyceridemia also developed quite frequently. Waist circumference increase was also prevalent among those who developed MetS during the follow-up period (data not shown). Of note, nearly one-third of the GDM women who developed new onset MetS in the year following initial testing had normal FBS.

**Table 3:** Results of Testing of Women with GDM Who Had No MetS at Initial Postpartum Testing, but Were Diagnosed with MetS by 12 Months, Displayed by Criteria and Composite Elements

	NCEP-ATP III Criteria		IDF Criteria	
	Number	%	Number	%
High density lipoprotein	0	0	2	14.3
Fasting blood sugar	13	72.2	11	78.6
Triglycerides	11	61.1	6	42.9
Blood Pressure	5	27.8	2	14.3
Body Mass Index (BMI)	8	44.4	0	0
Body Mass Index (BMI)/waist	0	0	1	7.1
Total	18	24.7	14/121	11.6

## Discussion

Gestational diabetes affects an estimated 7% of pregnancies in the United States and approximately 86% of those cases are GDM [9]. That amounts to approximately a quarter million cases each year [26]. The problem is increasing; the older age of women experiencing pregnancy and the increasing prevalence of both obesity and physical inactivity all contribute to this growth. The long-term health consequences of GDM may also be influenced by such factors [27].We found that over 25.5% of women with recent GDM experienced worsening of glucose tolerance in the first year following their deliveries; these rates that are higher than prior reports [22].

GDM is a marker of compromised pancreatic reserve, but also has been thought to represent insulin resistance [6, 28]. Stern et al. have described a “common soil hypothesis” underlying mechanism for the simultaneous increases in risks for both CVD and T2DM in women with GDM [29]. Whatever the underlying pathophysiology is, GDM is also closely associated with increased risk for early cardiovascular disease [3, 10, 30]. Our study shows that metabolic syndrome can frequently be diagnosed at the first postpartum visit; 24–31% of subjects had MetS diagnosed by of the prevailing diagnostic criteria. This may represent previously undiagnosed MetS, just as the postpartum oral glucose tolerance test can reveal previously undiagnosed diabetes, but it does underscore the fact that women with a history of GDM are at high risk for MetS and warrant testing. The prevalence of MetS in women who had experienced GDM in the last pregnancy continued to grow in the months following delivery. Another 12–25% of subjects developed MS by the end of the 12 months of follow-up. Some women diagnosed with MetS had normal fasting blood glucose levels, suggesting the need to monitor all women with prior GDM, not just those with DM.

The long term cardiovascular risks of women who have experienced gestational diabetes are very well documented [3, 6, 12, 13]. However, professional screening guidelines for women with a history of GDM focus almost entirely on initial postpartum and longitudinal tests of glucose tolerance, not for CVD [9, 15, 31–35]. Similarly, treatment recommendations for women with GDM are designed to delay the development of overt diabetes [36–41]. In particular, long term studies of potential lifestyle interventions make little or no explicit mention of other elements of metabolic syndrome or the risk factors for cardiovascular disease [42].

In most studies in which BMI and sometimes dyslipidemia were measured at different times following delivery, no formal analyses were performed for development of metabolic syndrome [22, 43]. Pallardo et al. found in the postpartum assessment that postpartum glucose intolerance was positively associated with abnormalities in BMI, waist circumference, waist-to-hip ratio; triglycerides and blood pressure, but not with total cholesterol or HDL cholesterol [44]. O’Higgins et al. reported 52% of postpartum women with prior GDM had dyslipidemia; 80% of women with abnormal postpartum oral GTT values had abnormal lipids [21]. Costacou et al. found an association between a history of GDM and excessive waist circumference [45]. Stuebe et al. and Meyers-Seifer found that women who had had GDM 3–5 years postpartum developed dyslipidemias at higher rates [46, 47]. O’Sullivan found that women with previous GDM followed for 22–28 years had increased risk for dyslipidemia, higher blood pressure and more abnormal electrocardiograms [48].

A few studies have specifically studied the association between a history of GDM and MetS in either the short term or with longer follow-up. At 3 months postpartum an association between GDM and metabolic syndrome was reported [49]. The incidence of MetS at 20 months was 37% compared to a 10% prevalence in controls [50]. At 8–10 years, women with history of GDM were 2–4 times more likely to have metabolic syndrome than controls; that risk was even higher among women with BMI > 30 kg/mL [51, 52]. Our study found even in a one-year follow-up that 10–25% of women with GDM in the most recent pregnancy who did not have MetS immediately postpartum developed it by 12 months.

This retrospective study has several limitations that may impact the generalizability of our findings. It reflects the experiences of subjects in one clinic. Our subjects were indigent, primarily Hispanic, women. Many potential candidates were excluded because they failed to return to the hospital-based clinic or they failed to follow-up for 12 months. Information about other variables that may increase cardiovascular risk, such as smoking, was not collected, but historically smoking was very rare in this population. Breastfeeding data over time were not collected, so no correction could be made for the impact of breastfeeding or stopping it can have on weight gain or other components of MetS. However, in previous studies of breastfeeding continuation patterns in women who delivered at Harbor UCLA, over half of women who said in labor that they planned to exclusively breastfeed had discontinued that practice by 6 weeks postpartum [53]. This means that the absence of this information would not likely have influenced our findings results greatly. Prior GDM or multifetal pregnancies were not controlled for; it was assumed that the impacts those factors might have had on the diagnosis of GDM would have resolved by the time of the postpartum testing.

While the high prevalence of MetS diagnosed at the time of postpartum testing following a GDM-complicated pregnancy may not represent causation, it certainly does identify a high-risk population in need of formal evaluation. However, considering the numbers of women who developed MetS by 1 year postpartum, guidelines for women with a history of GDM should include recommendations for assessment of cardiovascular risk factors. Those assessments should be at least as detailed for women with prior GDM as they are for women with PCOS who faced far lower risk of developing either overt diabetes or CVD. [54–67]. Active and ongoing monitoring for MetS among women with histories of GDM might motivate them to adopt lifestyle changes needed to prevent Type 2 diabetes as well as to reduce their risks for cardiovascular disease [37].

## Conclusions

Women who have experienced GDM represent a high-risk group that deserves formal evaluation over time not only for glucose tolerance, but also for cardiovascular disease risk factors. MetS is a convenient measure. Additional research is needed to help formulate guidelines to more thoroughly address the long-term cardiovascular health risks of women with a history of GDM.

## Abbreviations

ACOG: American college of obstetricians and gynecologists
ADA: American diabetes association
ATPIII: Adult treatment Panel III of the national cholesterol education program
BMI: Body mass index
CVD: Cardiovascular disease
GDM: Gestational diabetes mellitus
HDL: High density lipoprotein
IDF: International diabetes foundation
Met(S): Metabolic syndrome
PCOS: Polycystic ovary syndrome
T2DM: Type 2 diabetes mellitus
